# Current Perspectives in Cancer Immunotherapy

**DOI:** 10.3390/cancers11101472

**Published:** 2019-09-30

**Authors:** Theodoulakis Christofi, Stavroula Baritaki, Luca Falzone, Massimo Libra, Apostolos Zaravinos

**Affiliations:** 1 Department of Life Sciences, School of Sciences, European University Cyprus, 1516 Nicosia, Cyprus; christofi.theodoulakis@ucy.ac.cy; 2 Division of Surgery, School of Medicine, University of Crete, P.O. Box 2208, Voutes, 71003 Heraklion, Crete, Greece; vbaritak@gmail.com; 3 Department of Biomedical and Biotechnological Sciences, Oncologic, Clinic and General Pathology Section, University of Catania, 95123 Catania, Italy; luca.falzone@unict.it (L.F.); m.libra@unict.it (M.L.); 4 Epidemiology Unit, IRCCS Istituto Nazionale Tumori “Fondazione G. Pascale”, 80131 Napoli, Italy; 5 Research Center for Prevention, Diagnosis and Treatment of Cancer, University of Catania, 95123 Catania, Italy

**Keywords:** immune checkpoint blockade, anti-PD-L1, anti-PD-1, anti-CTLA-4, cytokine therapy, adoptive cell transfer, therapeutic vaccines

## Abstract

Different immunotherapeutic approaches have proved to be of significant clinical value to many patients with different types of advanced cancer. However, we need more precise immunotherapies and predictive biomarkers to increase the successful response rates. The advent of next generation sequencing technologies and their applications in immuno-oncology has helped us tremendously towards this aim. We are now moving towards the realization of personalized medicine, thus, significantly increasing our expectations for a more successful management of the disease. Here, we discuss the current immunotherapeutic approaches against cancer, including immune checkpoint blockade with an emphasis on anti-PD-L1 and anti-CTLA-4 monoclonal antibodies. We also analyze a growing list of other co-inhibitory and co-stimulatory markers and emphasize the mechanism of action of the principal pathway for each of these, as well as on drugs that either have been FDA-approved or are under clinical investigation. We further discuss recent advances in other immunotherapies, including cytokine therapy, adoptive cell transfer therapy and therapeutic vaccines. We finally discuss the modulation of gut microbiota composition and response to immunotherapy, as well as how tumor-intrinsic factors and immunological processes influence the mutational and epigenetic landscape of progressing tumors and response to immunotherapy but also how immunotherapeutic intervention influences the landscape of cancer neoepitopes and tumor immunoediting.

## 1. Introduction

During the last decade, immunotherapy has provided remarkable clinical responses to cancer patients. This turning point has significantly increased our expectations for successfully treating the disease [1]. Successful immunotherapies against melanoma, non–small cell lung cancer (NSCLC) and renal cell cancer have led to active clinical trials [2,3,4,5,6,7,8,9,10,11,12]. However, the percentage of responders is still rather low, underscoring the need to identify those patients who respond best to different immunotherapeutic approaches. Therefore, a suitable selection of cancer patients, based on their genetic profile along with other non-genetic determinants, seems to be critical in order to achieve the most successful therapeutic responses. The advent of next-generation sequencing, along with continuous technological advances in the field of sequencing, has provided us the ability to evaluate the required genomic information that we need in order to predict a patient’s response to immunotherapies. Here, we discuss the current immunotherapeutic strategies against cancer highlighting the pros and cons of each one. We also discuss the value of tumor neoantigens in the patients’ response to immunotherapy.

## 2. Immunotherapeutic Approaches against Cancer

Overall, there are four different immunotherapeutic strategies. These include immune checkpoint blockade, cytokine therapy, cellular therapy and therapeutic vaccines. To understand how immune checkpoint blockade works, it is critical to comprehend the constant interaction between tumor and immune cells in the continuous process of cancer development. Tumor cells exploit various immune-regulatory mechanisms to achieve immune escape, thus suppressing immune responses against them within the tumor microenvironment. This is more obvious as the disease progresses. Several immune-related cells work towards the establishment of an immunosuppressive microenvironment, including regulatory T cells (Tregs), dendritic cells (DCs), myeloid-derived suppressor cells (MDSCs) and regulatory B cells. Therefore, within the tumor microenvironment, cancer cells along with immune cells excrete inhibitory cytokines and express checkpoint inhibitors that dampen the anti-tumor activity of specific T cells. During the last decade, a rapid understanding of the mechanisms that most cancer cells use to hide from the immune system has led to the improvement of new, immunotherapeutic approaches against cancer. Especially, the use of anti-PD-1 or anti-PD-L1 monoclonal antibodies (mab) has yielded top-notch medical responses in several cancers. The most recent advances in cancer immunotherapy are summarized in Table 1 and discussed below.

### 2.1. Cancer Immunotherapy with Immune Checkpoint Blockade

#### 2.1.1. Monoclonal Antibodies against CTLA-4 and the PD-1/PD-L1 Axis 

The idea of immune checkpoint blockade involves the inhibition of the immune system’s intrinsic regulatory mechanisms, thus, driving the activation of a better anti-cancer immunological response. Since the recent encouraging clinical responses observed in patients who had been treated with mabs against the immune checkpoint proteins cytotoxic T-lymphocyte antigen-4 (CTLA-4) and programmed cell death (PD-1), the interest of the scientific community shifted in using such immune checkpoint blockade for treating cancer patients [63]. CTLA-4 is a membrane receptor on effector T cells that binds CD80/CD86 (B7.1/2) on antigen-presenting cells (APC) inducing the arrest of T-cells [64]. Likewise, PD-1 is another cell membrane receptor of activated T cells that interacts with PD-L1/2, inducing T-cell inhibition [65,66]. 

CTLA-4 blockade stimulates T-cells against cancer. Ipilimumab is an FDA approved anti–CTLA-4 mab against metastatic melanoma, that launched a new era in immunotherapy. We have witnessed a great progress since then, with the launch of novel immune checkpoint inhibitors, including two anti–PD-1 mabs, Pembrolizumab and Nivolumab. Although both mabs were initially approved against metastatic melanoma [67], NSCLC, renal cell carcinoma (RCC) [68,69], head and neck squamous cell carcinoma (HNSCC) after platinum-based chemotherapy [70,71,72] and refractory classic Hodgkin’s lymphoma (cHL), later on, they were approved for any unresectable or metastatic solid tumor characterized by mismatch repair deficiency (dMMR) or microsatellite instability (MSI+) [17]. This was the first time the FDA approved a cancer drug based totally on tumor genetics, instead of the tissue type or the location of the tumor. Such immune checkpoints could induce a durable clinical benefit, including a long-lasting regression of the tumor and an extended stabilization in patients with advanced NSCLC, melanoma and RCC [18,73,74].

Anti-PD-L1/2 targeting, has also attracted much interest in the clinical setting. We now know that PD-L1/2 expression on cancer cells or APCs suppresses CD8+ T cells (CTLs) and induces the function of T–regulatory (Treg) cells [66]. More than a few clinical trials have examined anti-PD-L1/2 alone or in combination with anti–PD-1 mabs [4,5,74,75]. 

Pidilizumab is another anti-PD-1 mab, tested in B cell lymphoma after autologous stem cell transfer and in relapsed follicular lymphoma, with 34% and 52% response rates, respectively [76,77].

In addition, the new anti-PD-L1 mab, Atezolizumab, showed low response rates (18%) in stage III–IV NCSLC and locally advanced and metastatic urothelial cancer (23%) [14,78,79]. In a recent comparison among these anti-PD-1 and anti-PD-L1 drugs, Nivolumab and Pembrolizumab were found to be related to increased objective response rates, compared to Atezolizumab and Nivolumab, which are correlated to fewer adverse events versus other drugs [80].

On the other hand, although Tremelimumab (anti-CTLA-4 mab) exhibited a good safety profile, it could not extend the overall survival of malignant mesothelioma suffering patients, versus the placebo [81,82]. Furthermore, blockade both PD-1 and CTLA-4 could reverse T cell suppression at both priming and effector cells [83].

At present, Pembrolizumab is the most successful immunotherapeutic approach for melanoma and other cancer types [84]. A phase III clinical trial compared anti-CTLA-4 and anti-PD-1 mabs and showed that Nivolumab-treated patients exhibited better response and survival rates compared to those treated with Ipilimumab. Patients treated with both checkpoint blockers, had better responses and survival [85]. In a similar study, combination therapy of Nivolumab and Ipilimumab on stage III/IV melanoma patients, yielded 57.6 objective response rates and 11.5% complete response rates, versus single therapies [86].

#### 2.1.2. Further Co-Inhibitory and Co-Stimulatory Markers

**Figure 1:**
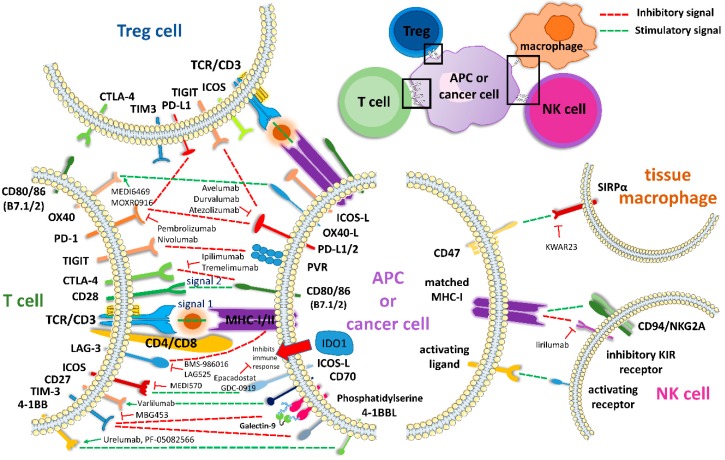
Immune-regulation within the tumor microenvironment is controlled by different checkpoints located on the T cell membrane. These, interact with their ligands found on the surface of antigen presenting cells (APC) or tumor cells, forming axes that provide either stimulatory signals (green) or inhibitory (red) signals between the two cells. Immune-therapeutic drugs belonging to checkpoint inhibitors act by blocking these axes (T).

Although the above-mentioned mabs gave encouraging results in metastatic patients, only a fraction of them are good responders. Lately, new approaches targeting further immune-regulating mechanisms are becoming available. These, comprise co-inhibitory and co-stimulatory immune receptors (markers) (Table 2). Such markers embrace T lymphocyte markers, macrophage markers and natural killer (NK) cell markers. Co-inhibitory signaling stem from the signals between TIGIT and PD-L1/2, PD-L1 and PD-1, TIGIT and PVR, CTLA-4 and CD80/86 (B7.1/2), LAG3 and MHCI/II, TIM-3 and Phosphatidylserine, TIM-3 and 4-1BBL or MHC-I and inhibitory KIR receptors. On the other hand, co-stimulatory signaling results from the interaction between OX40 and OX40L, CD28 and CD80/86(B7.1/2), ICOS and ICOS-L, CD27 and CD70, 4-1BB and 4-1BB-L, CD47 and SIRPa or MHC-1 and CD94/NKG2A, among other (Figure 1).

T lymphocyte markers include T-cell Ig and mucin-domain containing-3 (TIM-3) (previously recognized as hepatitis A virus cellular receptor 2, HAVCR2) [126], lymphocyte-activation gene 3 (LAG-3) [110], B7-H3 (CD276), B7-H4 (V-set domain-containing T-cell activation inhibitor 1, VTCN1), V-domain containing Ig suppressor of T cell activation (VISTA), cell surface transmembrane glycoprotein CD200 receptor 1 (CD200R), Inducible Costimulator (ICOS/ICOS-L), T cell immunoglobulin and ITIM domain (TIGIT), CD27/CD70 and glucocorticoid-induced TNF receptor (GITR). On the other hand, macrophage markers include CD47/signal-regulatory protein alpha (SIRPα) and indoleamine-2,3-dioxygenase (IDO1 or INDO); and NK cell markers include CD94/NKG2A and the killer immunoglobulin-like receptor (KIR) family (Figure 1).

Both TIM-3 and LAG-3 receptors are co-expressed on PD-1-expressing T cells, rendering them good candidates for combinatorial targeting with anti–PD-1 agents in PD-1 expressing tumors. TIM-3 is expressed on CD4+ Th1, Th17 and CD8+ CTLs and it is a T cell inhibitory receptor. TIM-3 is composed of a transmembrane domain, an extracellular glycosylated mucin domain and a variable immunoglobulin domain (IgV). TIM-3 has four relevant ligands, including Gal-9, HMGB1, Ceacam-1 and PtdSer. Gal-9 binding to TIM-3 induces an influx of Ca^2+^ to the intracellular region of Th1 cells, inducing T cell apoptosis. Binding of Gal-9, PtdSer and HMGB1 to TIM-3 functions as a positive regulator of dendritic cells (DCs), while that between Ceacam-1 and TIM-3 leads to a negative regulation of T cell responses [109,110]. Cells co-expressing TIM3 and PD-1, present more defects in cellular division and cytokine production compared to those expressing only PD-1 and blockade of both checkpoints can reverse T cell exhaustion [83]. TIM-3 blockade showed antitumor effects in many mouse tumor models and these effects were even greater once it was combined with CTLA-4 blockade [127].

LAG-3 is another co-inhibitory immune checkpoint [128] found on activated T, B, NK and plasmacytoid dendritic cells (pDCs) [124,129,130], where it predominantly binds to the antigen-MHC-II complex [131], negatively regulating T-cells [132] and activating Treg cells [126,128,133]. LAG-3 have four IgG domains with high structural homology with CD4 molecules while amino acid is <20% homologous to CD4. LAG-3 contains a unique “extraloop” in the membrane distal D1 domain, through which it binds to MHC-II molecules [134]. Combined PD-1 and LAG-3 blockade could reverse T cell function via different inhibitory pathways [83,126]. LAG-3-expressing Treg cells can effectively suppress cytotoxic T cells [135].

B7-H3 is a receptor found on the membrane of APCs, cancer cells and host cells, where it mainly suppresses T cell function. The anti-B7-H3 mab Enoblituzumab (MGA271) mediates potent ab-dependent cytotoxicity against various cancer types [136].

B7-H4 is a costimulatory membrane receptor expressed on APCs and cancer cells, interacting with the CD28 ligand and receptors on T lymphocytes causing an inhibitory effect. It has been associated with gastric carcinomas [137], breast cancer [138,139], non-metastatic clear cell renal cell carcinoma [140] and glioblastoma [141], among other tumor types. Its loss was also recently associated with the development of autoimmune diabetes [142]. 

VISTA is a checkpoint belonging to the B7 family. It is expressed on APCs and T cells in tissues with a significant number of infiltrating leukocytes [143] and it controls peripheral tolerance and anti-tumor immunity [120]. VISTA also suppresses T cell activation in a T-cell autonomous manner when expressed on CD4+ T cells. VISTA expression was found to be higher after Ipilimumab therapy in prostate cancer patients [144]. In NSCLC VISTA expression is associated with high number of tumor infiltrating lymphocytes (TIL load), PD-1, specific genomic alterations and patient clinical outcome [145].

ICOS is another transmembrane receptor expressed primarily on Treg cells but also in tonsillar T cells [91,146]. Its structure and function is similar to CD28 [91] and interacts with ICOS-L enhancing Treg cell function, T-cell proliferation, secretion of cytokines such as IL-10, IL-4, IL-5, IFNg and IL-17. The ICOS-ICOS-L interaction can also induce the excretion of antibodies by B cells. It also promotes the collaboration between T and B cells via the CD40/CD40L pathway [147]. ICOS expression is induced rapidly after T-cell activation [148]. A phase I clinical trial (NCT02520791) evaluated the anti-ICOS mAb (MEDI-570) in patients with relapsed/refractory peripheral T-cell follicular lymphoma and angioimmunoblastic T-cell lymphoma [149].

CD200R is another inhibitory glycoprotein expressed on the membrane of myeloid and lymphoid cells [150] and CD4+ T cells [151,152]. The CD200-CD200R pathway signals also stimulates the proliferation and activity of Treg cells and upregulates IDO activity [153,154]. The absence of the CD200R signaling was shown to inhibit the development of an endogenous tumor irrespective of the expression of CD200 [155]. A clinical trial using the humanized anti-CD200 mab, Samalizumab is on the way to treating metastatic bladder cancer [32].

TIGIT is found both on T cells and NK cells and it regulates T-cell mediated immunity via the CD226/TIGIT-PVR (poliovirus receptor or CD155) pathway [114]. TIGIT is mainly expressed on lymphocytes, including TILs and Tregs that infiltrate different types of tumors [156]. It has been shown to attenuate the immune response via direct signaling, inducing ligand signaling and competition with and disruption of signaling by the costimulatory receptor CD226 (also known as DNAM-1). PVR is also broadly expressed in tumors, suggesting that the TIGIT-PVR axis provides a major immune escape mechanism for cancer cells. Notably, TIGIT expression is tightly correlated with that of PD-1 and both are co-expressed on TILs. Combined blockade of both checkpoints could further increase cell proliferation, cytokine production and degranulation of both tumor antigen-specific CD8⁺ T cells and TILs in melanoma [157]. TIGIT could also synergize with TIM-3 to suppress an anticancer immune response [158].

Another co-stimulatory immune checkpoint of great interest is the TNF receptor family CD27. This binds to CD70 [159] regulating the activation of B-cells [160,161], mainly through the NF-κB and MAPK8/JNK pathways. Prolonged CD27-CD70 interactions have been show to exhaust T cells and induce lethal immunodeficiency [90]. Varlilumab is a new agonist CD27 mab that has been shown to activate T-cells in tumor models [33,34]. 

GITR is another co-stimulatory immune checkpoint molecule, encoded by the TNF receptor superfamily gene TNFRSF18 in humans. GITR expression is elevated upon T-cell activation and is critical in dominant immunological self-tolerance maintained by CD25+/CD4+ Tregs. GITR inhibits Tregs, extending the survival of effector T cells. High GITR levels have been reported on the surface of Tregs in mouse models but also on any activated T cells in humans, challenging its usefulness as a Treg marker [162]. Recently, PD-1 blockade and activation of GITR could synergistically induce the activation of T cells [163]. In addition, agonistic targeting of GITR was shown to augment TIL functionality in hepatocellular carcinoma [164].

The macrophage inhibitory marker SIRPα interacts with CD47, a broadly expressed transmembrane receptor, negatively controlling effector function of innate immune cells. The interaction of SIRPα with CD47 inhibits macrophage-mediated phagocytosis [165] similar to the self-signals provided by MHC-I molecules to NK cells via Ig-like or Ly49 receptors [166,167]. Cancer cells expressing high levels of CD47 could activate SIRPα and inhibit macrophage-mediated destruction. Engineered high-affinity variants of SIRPα antagonized CD47 on tumor cells and enhanced phagocytosis [168]. Furthermore, anti-SIRPα antibodies (e.g., KWAR23) were also found to help macrophages to diminish cancer progression and metastasis [35,36].

IDO1 along with IDO2 and tryptophan-2,3-dioxygenase (TDO), metabolize tryptophan to kynurenine, which is then metabolized to kynurenic acid [169,170]. Recent indication shows that IDO is upregulated in cancer progression, aiding tumor cells through pathogenic inflammatory processes to escape elimination by immune cells by inducing immune tolerance against tumor antigens [171,172]. IDO is expressed by some alternatively activated macrophages and other immune-regulatory cells [173], suppressing T cells and NK cells [174] and activating Tregs and MDSCs and promotes angiogenesis [171]. Contrarily, the pro-angiogenic vascular endothelial growth factor (VEGF) has been shown to increase the expression and activity of IDO in dendritic cells (DCs) thereby suppressing antigen-specific and mitogen-stimulated lymphocyte proliferation [175]. Moreover, the WNT signaling pathway and particularly the metabolic reprogramming of fatty acid oxidation (FAO) in DCs increases IDO expression while suppresses IL-6 and IL-12 cytokines; thus, aiding the tolerization of DCs within the tumor microenvironment and further contributes to anti-PD-1 immunotherapy resistance [176]. Targeting the WNT pathway with the specific tankyrase (TNKS) inhibitor, XAV-939, can enhance the immune response against pancreatic ductal adenocarcinoma (PDAC) cells with lymph node-positive metastasis [177]. IDO has been found to be upregulated in prostate cancer, colorectal cancer, pancreatic, cervical, gastric, ovarian, head, lung and other cancer types [178]. Epacadostat (INCB024360) combined with Pembrolizumab was recently announced to target IDO1 and provide encouraging antitumor activity in NSCLC patients and other tumors (ECHO-202/KEYNOTE-037) [31]. Navoximod (GDC-0919), a novel IDO1-specific checkpoint inhibitor, could also transiently decrease plasma kynurenine in patients with recurrent tumors. In this trial (NCT02048709), a response rate of 9% was reported [29]. The same checkpoint inhibitor, when administered with Atezolizumab in progressive solid cancers, exhibited activity but without a clear evidence for benefit of dual treatment [30]. Indoximod (previously termed as D-1MT) is an immunometabolic adjuvant recently shown to enhance T cell activity in cancer [179].

Nonetheless, a tight correlation exists between immune-infiltrate, angiogenesis and cancer progression and dissemination to distant sites and to nodal compartment. Indeed, CD8+ T cells and immune cells come and go across the permeable capillaries. Because of these intimate interactions, it happens that local dendritic cells (DCs) within the tumor microenvironment become tolerant to immunotherapeutic strategies and thus, promote immune evasion and immunotherapy resistance. One such mechanism was described in melanoma by Zhao F et al. [176], where blockade of the Wnt5a-β-catenin-peroxisome proliferator-activated receptor-γ (PPAR-γ) signaling pathway augmented anti-melanoma immunity, enhanced the activity of anti-PD-1 antibody immunotherapy and suppressed disease progression in a transgenic melanoma model. Increased WNT activation and a peculiar immune microenvironment were also shown to increase the likelihood of lymphatic dissemination in pancreatic ductal adenocarcinoma (PDAC) [177]. In this study, regional lymph node positive PDACs were enriched in M2 macrophages and activated DCs [177]. Furthermore, endothelial cells (ECs) also interfere with the efficiency of DC maturation. VEGF was found to increase the expression and activity of IDO in DCs, having a suppressive effect on Ag-specific and mitogen-stimulated lymphocyte proliferation [175].

CD94/NKG2 is a dimer between a C-type lectin receptor (NKG2) and the CD94 molecule, primarily expressed on NK and a few CD8+ T cells [180,181]. Activating CD94/NKG2 receptors stimulate cytotoxic activity of NK cells, whereas inhibitory receptors impede it [182]. The CD94/NKG2 dimer recognizes non-classical MHC-I glycoproteins [183]. NKG2A and NKG2B receptors transmit inhibitory signals through their Immunoreceptor Tyrosine-based Inhibitory Motif (ITIM) tails, while NKG2C, NKG2D, NKG2E and NKG2H are activating receptors.

KIR receptors are membrane glycoproteins found on NK cells and T cells [184,185], suppressing their cytotoxic activity via inhibitory signals that they produce [186]. Only a few KIR receptors are activating, showing that their recognition of MHC molecules triggers the cytotoxic activity of their cell [187]. Recently prolonged KIR blockade using the anti-KIR2D mAb, lirilumab, was announced to be safe and well tolerated [188]. Nevertheless, the EffiKIR clinical trial (NCT 01687387) failed to show clinical effects on AML patients [189].

#### 2.1.3. Adverse Effects Associated with Checkpoint Blockade 

Inhibition of immune-checkpoint receptors using the above-mentioned therapeutic mabs for treating cancer patients is linked to many side effects of various rates, which resemble autoimmune reactions [190]. Furthermore, despite many cancer patients respond well to immune checkpoint blockade, a significant proportion of these tumors will eventually develop tumor resistance and will progress. Therefore, finding the proper balance between immune-therapies and/or finding new therapeutic tools to overcome such a resistance will significantly improve the clinical outcome [191]. 

In particular, the rate of incidence and the types of adverse events related to immune-checkpoint inhibitors depend on the specific features of patients and on the type of agent used [192]. Several studies demonstrated that the checkpoint inhibitors are more tolerated compared to classical chemotherapeutic agents; in particular it was demonstrated that PD-1/PD-L1 inhibitors have less toxicity compared to the anti-CTLA-4 antibody Ipilimumab [193]. Overall, the most common immune-checkpoint inhibitors-related toxicity are systemic, endocrine, dermatologic and gastrointestinal side effects [192,193]. Regarding the systemic adverse events, these encompass fatigue and fever, asthenia, hypotension, dyspnea and other symptoms due to infusion reactions whose incidence is about 25% of patients treated with immunotherapy [16,194]. Other studies paid the attention to the endocrine, dermatological and gastrointestinal adverse events showing that treatments with Ipilimumab, Nivolumab and Pembrolizumab were able to induce skin toxicities due to allergic reaction to the compounds [195]. Ntali and colleagues (2017) have made a careful review of the literature regarding the endocrine sequelae of cancer immunotherapy, showing that these treatments may lead to hypophysitis, adrenalitis and both hypothyroidism and hyperthyroidism depending on specific characteristics of patients [196]. Finally, as observed for several chemotherapeutic agents, also the administration of immune checkpoint inhibitors is correlated to several gastrointestinal side effects mainly including diarrhea, colitis, nausea, vomiting and abdominal pains [197]. Furthermore, adverse effects including rash or pruritus, thyroiditis, hypothyroidism or hypophysitis and asymptomatic pancreatitis were reported in dMMR cancer patients treated with PD-1 blockade [198].

### 2.2. Cancer Immunotherapy with Cytokine Therapy

The tumor microenvironment (TME) consists of cancer cells and stroma including non-malignal endothelial, fat, blood and immune cells of the surrounding niche [199]. Tumor progression is depended on the interaction of cancer cells with their microenvironment, influencing cancer cell survival and growth, invasion and metastasis, through an orchestrated signaling crosstalk that drives tumor evolution [200]. There, tumor-associated neutrophils (TANs), tumor-associated macrophages (TAMs), innate lymphoid cells (ILCs), MDSCs, mast cells, T cells and NK cells produce various factors such as enzymes, chemokines and cytokines which can increase angiogenesis but also modulate the local immunity and establish immunosuppression [201,202,203].

Cytokines are molecules that promote inter-communication between the immune cells and were initially used as an immunotherapeutic approach. Proinflammatory cytokines stimulate crucial immune effectors, including T cells and NK cells. In the tumor’s microenvironment, cytokines mediate cancer progression in solid and hematologic tumors. For example, in multiple myeloma several cell types within the bone marrow, including cells of the immune system, mesenchymal stem cells and bone marrow stromal cells, can contribute to the development of myeloma bone disease [204]. The cytokine- and cell-adhesion-dependent bone marrow niche and stromal microenvironment support the formation of new vessels and the proliferation of multiple myeloma, irrespective of immune-surveillance [205,206,207,208]. Leone et al. provided evidence that the intimate interaction between endothelial cells, tumor cells and CD8+ T cells creates a permissive immune-microenvironment within the bone marrow that allows undisturbed cancer proliferation [209]. They demonstrated that endothelial cells act as APCs, stimulating a central memory CD8+ T cell population, which negatively regulates the effector memory CD8+ T cells with anti-tumor activity. Remarkably, a defective immunosurveillance allows for the persistence and proliferation of multiple myeloma cells—an immune-microenvironment disease evolution characterized by exhausted CD8+ cells, overexpressing check point molecules such as LAG3 and PD1, in preclinical models offers suitable targets for increased survival in in vivo models [205,206,210]. In a clinical setting, a patient with a larger CD8 cytokine profile, along with competent CD8 T cells and dendritic cells had an increased overall survival and time to progression [206,211]. Therefore, it is likely that angiogenesis within the bone marrow, a recognized hallmark of multiple myeloma progression, parallels multiple myeloma evasion from T cell immune surveillance [208,209,212].

Importantly, cancer-associated fibroblasts (CAFs) are also implicated in mediating tumor-promoting inflammation by secreting cytokines and chemokines that mediate the recruitment and activation of immune cells and by their reciprocal interactions with immune cells in the tumor microenvironment [213,214]. CAFs also contribute to immune escape by upregulating immunosuppressive cytokines and immune checkpoint ligands, as well as via the exclusion of anti-tumor CD8+ T cells from cancer cells and by affecting the functional differentiation of tumor infiltrating inflammatory cells [215]. Furthermore, the intimate interaction between endothelial cells, tumor cells and CD8+ T cells creates a permissive immune microenvironment that allows undisturbed cancer proliferation [209].

Thus far, two cytokines are FDA approved as anti-cancer therapeutic agents—IL-2 against metastatic melanoma and kidney cancer and IFN-α as adjuvant therapy against stage III melanoma. IFN-α is an all and more preferable treatment for diseases like Philadelphia-negative myeloproliferative neoplasms (MPNs), essential thrombocytosis, polycythemia vera (PV) and primary myelofibrosis (PMF) [216,217]. Nevertheless, IFNα treatment has been linked with side effects, even at low-doses and of its pegylated form [42,217,218,219,220]. Among the major pathways by which IFN-α2 acts, is the JAK/STAT signaling pathway [221,222,223,224]. IFNα2 has anti-proliferative, pro-apoptotic, antiangiogenic and immunomodulatory mechanisms of action [221,222,224,225] and it can also downregulate telomerase reverse transcriptase and telomerase activity [226].

Modern approaches can also interfere with cytokine-related immune effect. For example, cytokine production was shown to be induced by CAR-T cells, such as HER2-specific designed ankyrin repeat protein (DARPin28z) CAR T-cells, upon antigen binding [227]. Furthermore, the multi-specific DARPin^®^ molecule, MP0250, was shown to strongly neutralize VEGF and HGF causing a significant reduction in the number of vessels and was defined as a novel combination drug for treatment of multiple myeloma patients [228]. Profiling for angiogenesis related cytokines of endothelial cells isolated from bone marrow of multiple myeloma patients treated with MP0250, confirmed that neutralization of VEGF and HGF results in a change of the cytokine profile towards in favor of antiangiogenic acting cytokines [228].

### 2.3. Cancer Immunotherapy with T-Cells Redirected against Tumor Antigens

Many hopes have been put on the adoptive cell transfer (ACT) technology for treating B cell malignancies among many other cancer types. In ACT, autologous TILs are initially isolated from an existing tumor mass, co-cultured with IL2 to grow ex vivo [229] and subpopulations of these proliferating cells are tested in vitro against the patient’s original tumor. Following, the number of cells that are active against the tumor is increased [18,73]. Then, the high numbers of TILs are reinfused to the patient to provoke in vivo immune response. In fact, ACT has produced major clinical response in metastatic cancer patients [230,231,232]. Although this technology could be used against different types of cancer, efficacy has been shown only against a few types, including melanoma, possibly related to its immunogenicity and its higher affinity for immune clearance. Therefore, the interest has now shifted into the development of ACT strategies using modified patient-specific T cells that carry genetically engineered antigen receptors in the form of either native or recombinant TCRs or chimeric molecules (CARs) [233].

#### 2.3.1. TCR Engineered-T Cell Immunotherapy 

In addition to TIL-based immunotherapy where patient’s TILs are activated and expanded ex vivo, following re-infusion into the patient’s blood circulation, patient’s T lymphocytes can also be genetically engineered to express TCRs obtained from tumor-reactive T cell clones, thereby creating in vivo large quantities of tumor reactive T-cell populations [234]. Given that naturally existing TCRs recognize processed peptides presented on MHC, the TCR engineered-T cells may target a large number of both surface and intracellular tumor-associated antigens. However, TCRs investigated thus far for ACT, have been limited to mostly MHC-I-restricted candidates [235]. Efforts to better engineer high-affinity TCRs have yielded recombinant TCR-carrying T cells that are better effectors than endogenous tumor reactive T cells [235]. The list of ongoing clinical trials using TCR-engineered T cells is progressively expanding, with most of them directed against well characterized tumor-expressed antigens such as gp100 and tyrosinase for melanoma, WT1 for AML and NSCLC, HPV-6 for HPV-associated cancers and others [235,236]. 

Although the TCR-engineered-based ACT strategies remain an exciting and rapidly evolving field, limitations on the treatment efficacy and the number of patients that can be treated still exist. The MHC restriction especially this of HLA class I molecules which are often downregulated in cancer cells, the dependence on co-receptors such as CD8 and the competition between the transgene receptor and the endogenous TCR for interaction with the available CD3 complex components, usually minimize the therapeutic effectiveness of the approach [236,237]. In addition, the presence of endogenous TCR may allow for chain mispairing between endogenous and introduced TCR a and β chains, leading to reduced receptor expression and often to serious adverse effects propagated by the unpredictable generation of self-reactive TCRs [238,239]. Currently, such “off target, off tumor” effects as the result of TCR chain mispairing or high affinity TCRs recognizing “off target” epitopes, are alleviated by several strategies including among others, codon optimization of the TCR a and β genes and the use of single chain recombinant TCRs [240,241]. 

#### 2.3.2. CAR Engineered T-Cell Immunotherapy versus T-Cell Activating Polyspecific Antibodies 

**Figure 2:**
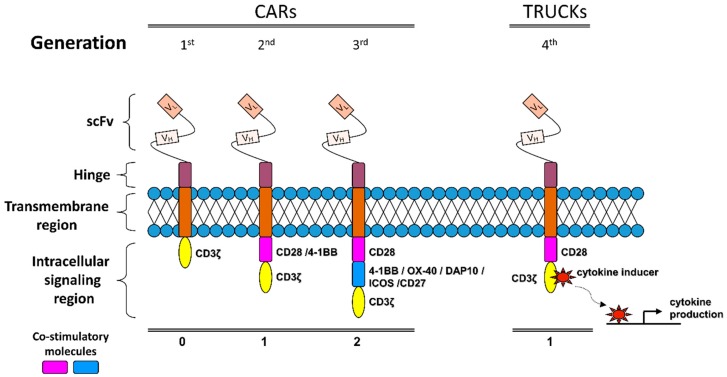
Motifs of chimeric antigen receptor (CAR) constructs. The basic structure of CARs (1st generation) contains extracellular mAb-derived variable heavy (V_H_) and light (V_L_) chains directed against a native tumor-specific antigen (extracellular domain) fused with a TCR CD3ζ chain-containing intracellular signaling domain through a transmembrane linker. Second and third generation CARs provide an improved receptor signaling strength and persistence by incorporation, in the basic CAR structure, of one or two co-stimulatory molecules, respectively. T-cells redirected for universal cytokine mediated killing (TRUCKS) serve as the 4th generation of chimeric antigen receptors constructed on the base of the 2nd generation of CARs with the addition of a NFAT-driven cytokine producing gene cassette, such as IL-12. scFv, single-chain variable fragment.

Unlike the engineered TCRs, chimeric antigen receptors (CARs) are “synthetic molecules” and they do not exist naturally [242]. CAR technology was initially introduced 25 years ago via T cells transduced, by a disarmed virus, to express a chimeric antigen receptor that consists of an extracellular mAb-derived single-chain variable fragment (scFv) region and various intracellular signaling domains. A CD8 or IgG4-derived hinge-transmembrane fragment is used to connect the intracellular and extracellular domains of CAR [243]. Depending on the number of the co-stimulatory domains included in the cytoplasmic fraction of the receptor, together with the CD3ζ TCR constant signaling region, the CARs can be classified into 1st, 2nd or 3rd generation receptors [235] (Figure 2). 

Encouraging early clinical results with second-generation anti-CD19 CARs were observed in patients with lymphoma [244,245]. However, the high affinity for target cells conferred by the Ig component of CARs, combined with amplified non-physiologic T-cell signaling in 2nd and 3rd -generation constructs, has been associated with serious adverse events [246]. Reducing on-target toxicities while maintaining antitumor efficacy is an important goal of current investigations [247].

The incorporation of co-stimulatory domains optimizes not only the production of the cytokines necessary for the proliferation of the CAR T cells but also contributes to the more efficient and long-lasting signal transmission by the receptor, thus overcoming most of the immune checkpoint-derived restrictions [248]. 4-IBB, CD28, DAP10, ICOS or OX-40 are within the list of the most commonly utilized co-stimulatory molecules for the construction of the 2nd and 3rd generation CARs. Both generations have shown in vitro and in vivo good efficacies on T cell function following antigen stimulation in multiple malignancies [249,250,251,252]. 

As CAR technology is moving forward, a 4th generation of chimeric antigen receptors has been recently constructed on the base of the 2nd generation CARs with the inclusion of a cytokine producing gene cassette driven by an NFAT sensitive promoter (Figure 2). These chimeric receptors, widely known as T-cell redirected for universal cytokine mediated killing (TRUCKs) are able to better facilitate and sustain T cell activation compared to alternative and potentially less toxic but effective approaches, namely Bispecific T cell engagers (BiTEs), Tri-specific T cell engagers (TiTEs) and Dual-affinity Re-targeting Antibody (DART) molecules. TRUCKs manage to do so, while attracting innate immune cell populations in order to increase receptor specificity by eliminating antigen-negative cancer cells in the targeted microenvironment [253]. 

Ideally, CAR T cell therapy could redirect T cell killing to cells that express the antibody’s cognate tumor associated surface antigen, in a MHC-independent manner [254]. Since 2017, CARs have been in the lead against hematologic malignancies, including chronic (CLL) and acute (ALL) leukemia [255,256,257], as well as relapsed/refractory multiple myeloma (MM) [251]. Most of the FDA approved CARs or currently in clinical trials, are directed against B-cell restricted antigens, including CD19, CD138, kappa-light chain and B cell maturation antigen (BCMA) [251,258,259]. To date, the administration of CAR T-cell immunotherapy in hematologic cancer patients has yielded encouraging results with the clinical outcomes to depend mostly on the nature of the targeted antigen, the CAR construct, the dose used and the disease stage [251,260]. On the other hand, CARs designed to treat solid malignancies are still in an infant phase and only recently have yielded some promising results [258,261,262]. Currently, in clinical trials are 2nd and 3rd generation CARs designed to target a wide variety of antigens expressed on solid tumors, including among others the HER2 and EGFR-positive malignancies, carcinoembryonic antigen (CEA) for metastatic tumors of various origin, PCMA antigen for prostate cancer, EGFRvIII for glioblastoma, GD2 and Li-CAM for neuroblastoma, VEGFR-II and cMet for melanoma, Muc-1 for various malignancies and GPC3 for hepatocellular carcinoma [235,262,263]. Ongoing clinical trials using 4th generation CARs (TRUCKs) are directed against PSMA and GD2 in bladder and neuroblastoma cancers, respectively [263]. Currently the CAR technology is moving forward and multi-specific designed CAR T cells can address heterogeneous tumors [263].

Although CARs provide MHC- independent antigen recognition and lack of restrictions related to endogenous co-stimulation of the receptor, the high affinity for target cells conferred by the Ig component of CARs, combined with amplified non-physiologic T-cell signaling in 2nd and 3rd-generation constructs, have been associated with serious adverse events [246,264]. The so called “on target, off tumor” phenomenon which is translated in effective cytotoxic action of CAR-T cells against non-tumor cells that express the targeted antigen, has been noted in most CAR applications resulting in significant limitations of their tumor-specific effectiveness [246,247,264]. In addition, CAR recognition is only limited to surface antigens, while targeted antigen loss, anti-transgene immunogenicity especially against murine-derived scFv or activation of immune-checkpoint pathways such as PD-1/PD-L1 by MDSCs can further decrease the efficiency of CAR-T cell based ACT trials [235,265]. Other serious adverse effects include but are not limited to, cytokine storm [266] and tumor lysis syndrome [264], suggesting that continuing efforts are required to decrease the toxicities related to CAR-T cell immunotherapeutic applications and improve the clinical outcomes. 

New approaches by bispecific antibodies and antibody-associated immune modulation can now offer intriguing opportunities, as discussed in Reference [267]. On this axis, intriguing opportunities for redirecting T cells against cancer cells have been offered by the development of alternative antibody-associated immune-modulating approaches, including polyspecific antibodies such as BiTEs, TiTEs and DART molecules [267,268]. Polyspecific antibodies can be directed against multiple tumor antigens to eradicate tumor cells more precisely and effectively [269].

Bispecific antibodies with ability to engage two different antigens are the most commonly used polyspecific antibodies [270]. BiTEs, also known as T-cell activating bispecific antibodies (TABs), are usually composed of two tandem single chain variable fragments (scFvs), each with a unique antigen specificity [268,270,271,272]. Most BiTEs, including Blinatumomab, the only bispecific antibody approved for the treatment of ALL, simultaneously recognize CD3 and a tumor-associated antigen (TAA), thus enabling engagement of effector T cells with cancer cells [268,269,273]. This crosslinking leads to T cell activation and direct cytotoxicity regardless of TCR specificity and MHC restrictions, while induction of cytokine responses may further trigger a bystander effect on adjacent cells [268,274]. DART proteins, are even smaller than BiTEs and have a diabody format where one V_L_ chain is followed by the V_H_ chain of the second binder and the two polypeptide chains align in a head-to-tail fashion [268].

Although BiTEs may redirect the large reservoir of resident T cells to tumors, while other T-cell activating approaches, such as CARs or TRUCKS rely on significant in vivo expansion to exert antitumor activity, it is still unclear whether BiTEs may potentially have a comparative advantage over CARs, with respect to their toxicity, specificity and binding activity on the target epitopes [275]. Among the major limitations in the activity and effectiveness of BiTEs appear to be T cell anergy and exhaustion triggered mainly by PD1/PD-L1 activation [268]. Efforts to bypass the aforementioned limitations by combinations of BiTEs with antibodies against immune checkpoint inhibitors (checkpoint blockage) or other molecules, have already achieved promising preclinical results; however further research is needed to validate the efficacy of the method and the durability of the results for most BiTEs [268]. Moreover, and contrary to the considerable activities of most BiTEs on hematologic malignancies—such as ALL—the therapeutic benefit of newly developed BiTEs on solid tumors is still under investigation [269]. Mechanisms associated with TAB’s failure to kill solid tumors mainly include, antibody inability to access the target tissue, dose-limiting toxicities and low half-lives, on target but off-tumor toxicities, fatal cytokine release syndrome and other adverse effects similar to those observed with CAR-T cell approaches, as described above [268]. DARTs also face the problem of low in vivo half-life, which can be partially solved by fusion of an Fc domain that prolongs antibody stability in the serum [268,276]. However, the two linkers on the diabody format may reduce the mobility of the antigen binding sites, thus restricting antigen recognition [277]. 

Complimentary to T-cell activating polyspecific antibodies, innovative techniques have also introduced BiKEs and TriKEs, in an effort to enhance the therapeutic benefit of NK cell-based immunotherapy. BiKEs and TriKEs directly trigger NK activation and cytotoxicity against various TAAs on tumor cells after engagement to CD16 [269,278,279]. Although in vivo safety is still uncertain for those molecules, they keep high promise for use in triggering cytokine production [280].

As discussed by Rancie et al., there is no doubt that new generation polyspecific T and NK-cell engagers have successfully joined CARs on the cancer immunotherapy highway [280]; however, major differences between the two strategies determine not only their performance but also their suitability for use depending on the type of cancer and the targeted TAA [269]. Although CAR T cell production is significantly costly, as it is patient specific, however it appears that new CAR T cell constructs have the comparative advantages of being capable to (1) bind TAAs even at low density causing direct lysis, (2) naturally extravasate and travel between endothelial barriers, thus allowing better distribution and (3) expand in vivo and establishing a memory cell pool that facilitates prolonged response [269] Overall, efforts to enhance the therapeutic benefit of CAR-T based approaches by developing strategies to reduce the adverse effects and especially on-target toxicities, while maintaining antitumor efficacy, are important subjects of intense research on CAR-T cell immunotherapy era [247].

## 3. Therapeutic Vaccines

Vaccines against tumor initiation-involved viruses can provide a precautionary measure against tumor development. The human papilloma virus (HPV) is linked to cervical and oropharyngeal head and HNSCC and vaccination with a recombinant vaccine against certain subtypes of HPV can now decrease the occurrence of these cancers [56,57,71,281]. Likewise, vaccination against hepatitis B virus (HBV) can help prevent hepatocellular carcinoma [55]. 

Apart from preventative vaccines, therapeutic vaccines against tumor antigens can also yield encouraging immunotherapeutic outcomes. Engineered vaccines against both tumor-specific antigens (TSAs) and tumor-associated antigens (TAAs) can provoke immunological response against cancer cells, which persists in the future with the help of memory T and B cells. So far, the only FDA approved therapeutic cancer vaccine, is Sipuleucel-T, administered in prostate cancer patients. Autologous lymphocytes are isolated from the patient and exposed in vitro to a conjugate of prostatic acid phosphatase with granulocyte-macrophage colony-stimulating factor (PAP-GM-CSF), before being reinfused to the patient. These immune cells have mainly differentiated into DCs and present the PAP antigen, which is found in the majority of the prostrate cells, to T cells provoking an immune response [59]. Sipuleucel-T increased prostate cancer patients’ overall survival by 4 months and provided better survival rates versus the controls [59]. A trial using a vaccine against gp100 combined with immune-modulators (e.g., IL2) is under way for melanoma patients [60]. 

In addition, stimulator of interferon genes (STING) is a transmembrane protein that induces type I interferon production in cells infected with intracellular pathogens. Combination therapy of STING with anti–PD-1 could promote antitumor innate immunity and improved response [61]. 

GVAX is another therapeutic vaccine against pancreatic cancer. It is composed of whole tumor cells that have been genetically engineered to express GM-CSF. Initial trials with GVAX did not improve the overall survival of patients with metastatic pancreatic cancer but when combined with the mesothelin-secreting vaccine, CRS-207, it synergistically produced longer overall survival [62]. Nevertheless, the incapability of most vaccines to produce immune response, when administered alone, shows the ability that tumor cells have to evade the immune system and the necessity for successful combinatorial therapies. As most of the therapeutic vaccines have yielded modest clinical benefit to patients with advanced cancers, their value is now underscored.

Additionally, the introduction of personalized mRNA/DNA-based vaccines against TSAs of the patient, provides the prospect for better anticancer vaccines.

A novel approach suggests that the immune system can neutralize various immune-suppressive signals, through self-reactive, pro-inflammatory T cells which can target inhibitory Treg cells and were therefore called anti-regulatory T cells (anti-Tregs) [282]. The activation of these anti-Treg cells was suggested to offer a new path to block immune inhibitory pathways within the tumor microenvironment [283]. Thus, if successfully targeted, a therapeutic vaccination aiming to activate anti-Tregs could enhance anti-tumor immunity by relieving immune suppression and potentiating effective anti-tumor T cell responses [284]. The potential use of an anti-Treg-activating vaccine was recently suggested that it could attract T cells into the tumor microenvironment, eventually producing more susceptible targets to immune checkpoint inhibition [284].

## 4. Mutational and Epigenetic Landscape in Tumor Progression and Response to Therapy 

The genetic code of cancer cells includes loss- and gain-of-function mutations, derived from DNA replication errors or caused by genotoxic agents [285,286]. These mutations can be inherited or acquired (germline or somatic) in pre-malignant cells and provide the stage for further genetic alterations, driving cancer pathogenesis [287,288]. Several theories try to explain the mutational landscape evolution of tumors [289,290,291]. Been widely adopted, the clonal evolution concept suggests that driver mutations are positively selected based on their ability to provide a fitness advantage to tumor cells, such as promoting survival and growth, where they clonally expand in expense to less fit tumor cells [289,292]. On average, tumors carry 4 coding substitutions in driver genes (ranging from 1 to 10 depending on the tumor type) half of which are not in known cancer genes [293]. Negative selection is mostly absent in cancer genomes with less than 1 coding substitution in a given gene, as the majority of these mutations lead to cell death and are eventually lost [293]. Moreover, neutral or passenger mutations dominate cancer genomes but are predominantly synonymous with no selective advantage. However, some synonymous mutations, such as the T125T hotspot mutation in *TP53*, can affect gene splicing but also protein folding and act as driver mutations [293,294,295,296].

Besides exogenous mutagens, cell intrinsic processes and especially DNA replication errors is the primary source of genomic instability and ultimately malignancy [297]. DNA polymerases and mismatch repair (MMR) proteins are responsible for correcting DNA replication errors thereby maintaining genomic stability [298]. Mutations in these proteins significantly impair DNA replicative fidelity, frequently resulting in microsatellite instability (MSI) [299,300,301,302,303]. 

**Figure 3:**
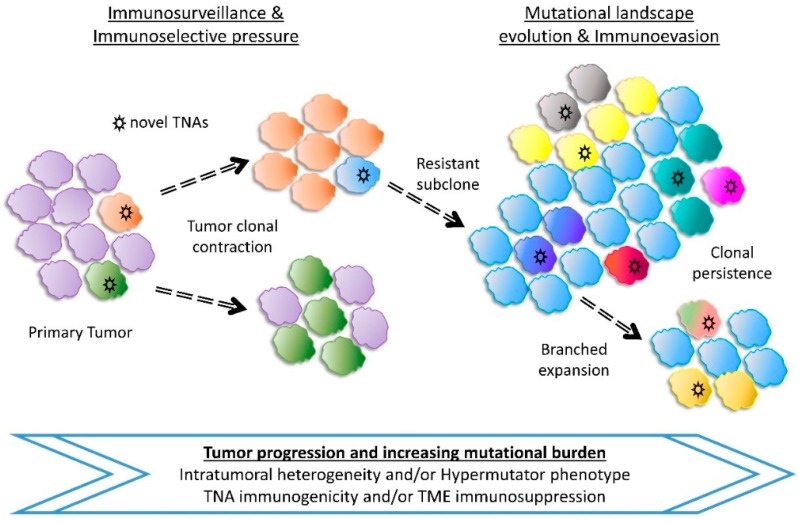
Clonal evolution of tumor progression and tumor neoantigens (TNA) landscape diversity. Cancer pathogenesis is initiated by driver mutations that provide a fitness advantage to cells which evolve into the primary tumor. Additional mutational changes drive tumor evolution and are influenced by intrinsic and extrinsic factors. Regarding immunity, immunotherapeutic intervention and/or host immunosurveillance exert a selective pressure to immunogenic clones leading to the rise of resistant subclones. These tumors can harbor TNAs that are recognized as self and elicit poor immunological responses or induce local tumor microenvironment (TME) immunosuppression. Persistent tumors expand and acquire more mutations leading to intratumoral heterogeneity (ITH) and/or hypermutation, further affecting the TNA repertoire.

The mutational landscape of tumors can shape cancer immunity and response to immunotherapy and other chemotherapeutic strategies [304] (Figure 3). Tumor neoantigens (TNAs) are generated by nonsynonymous mutations in malignant cells, providing a novel target for immune responses [305,306,307,308]. Some neoantigens can be immunogenic and induce host immune responses against tumors. On the other hand, a high mutational burden and the number of TNAs can exhibit adverse effects and favor immunoevasion of cancer cells from host immune responses [309,310]. Moreover, these hypermutated tumors can also induce immunosuppression by expressing immunoinhibitory molecules like PD-L1 and CTLA-4, thus developing adaptive resistance [310,311,312]. The tumor microenvironment, TNA immunogenicity, intratumoral heterogeneity (ITH) and other factors seem to play a role on how tumors respond to immunotherapy [313,314,315]. The immunophenotype is also a key determinant that drives clinical decision in cancer and is now considered an additional challenge for immunotherapy. For example, Atezolizumab (anti-PD-L1) has shown encouraging activity against metastatic colorectal cancer when combined with chemotherapy and/or targeted therapies, especially with MEK inhibitor Cobimetinib [316]. Similarly, combination therapies of immunotherapy with existing chemotherapy, radiation or other immunotherapy with different mechanisms of action has been suggested to be evaluated to achieve excellent outcomes in patients with esophageal cancer [317].

Interestingly, the number of TNAs is highly correlated with the mutational load and the infiltration of TILs [318]. Furthermore, predicted immunogenic mutations in cancer patients extracted from The Cancer Genome Atlas (TCGA) database, linked mutational epitopes with higher tumor CTL content and increased patient survival [319]. The positive prognostic value of elevated mutational and TNA burden is also prevalent in a variety of cancers that show active immunological responses and signatures, regardless of treatment that are associated with improved survival of cancer patients [313,320,321,322,323,324]. The recognition of TNAs by host T cells makes them ideal immune targets and gives hope for further immunotherapeutic intervention by targeting them [325,326,327].

In addition, several studies demonstrated that the evaluation of the epigenetic status of patients, as well as induced epigenetics modification, might predict the response rate of patients and boost the efficacy of cancer immunotherapies [328,329]. Several studies focused the attention on the role of methylation events and microRNAs (miRNAs) alterations in cancer development, aggressiveness and response to therapies [330]. In particular, the methylation status of PD-L1 may be associated with the survival rate of acute myeloid leukemia patients, where patients with hypomethylation of PD-L1 promoter have a lower overall survival compared to patients with high levels of methylation [331]. Other studies demonstrated that the treatment with hypomethylating agents before the administration of immune checkpoint inhibitors are able to improve the response rate in several tumors [332,333,334]. 

Regarding miRNAs, numerous studies have identified specific miRNAs associated with the development, progression and response to therapies in different cancer types [335,336,337,338,339,340,341]. In particular, it was demonstrated that miRNAs may serve for the development of new diagnostic, prognostic and therapeutic strategies in the context of cancer immunotherapy. Boeri M et al. (2019) identify a set of miRNAs whose expression levels were associated with the response rate of NSCLC patients to PD1/PD-L1 inhibitors [342]. Furthermore, in a recent review of literature it was reported that several miRNAs, of which miR-34a and miR-200 represent two of the most representative miRNAs, are able to directly regulate the PD-L1 expression leading to a poor prognosis of mesothelioma patients [343]. Despite the growing body of evidences about the assessment of the mutational and epigenetics landscape of patients to predict the response to immune checkpoint inhibitors, further studies are needed to identify specific biomarkers and clarify the functional roles of genetic and epigenetics modification during cancer immunotherapy.

## 5. Modulation of Gut Microbiota Composition and Response to Immunotherapy

In the last decade, several studies demonstrated that the human gut microbiota is involved not only in gastrointestinal diseases but play also crucial roles in the development and progression of other pathological conditions, including neurodegenerative diseases and cancer [344]. In particular, it was demonstrated that the composition of human gut microbiota may influence the response rate of cancer patients treated with chemotherapy and immune checkpoint inhibitors enhancing or reducing the overall survival and progression-free survival of patients [345,346,347]. With a particular regard to immunotherapy, it was demonstrated that the modulation of gut microbiota with the administration of specific probiotics strains, including *Lactobacillus rhamnosus* GG, *Bifidobacterium longum*, *Enterococcus faecium* and *Collinsella aerofaciens*, may influence the patient’s response to anti-CTLA-4 and anti-PD-1/PD-L1 inhibitors [346,348,349].

To further strengthen the important role of gut microbiota homeostasis during immunotherapy, other studies demonstrated that antibiotic treatments before the administration of immune checkpoint inhibitors lead to a lower response rate to immune checkpoint inhibitors [350]. 

Finally, it was also demonstrated that microbiota modulation through fecal microbial transplantation (FMT) could be a good strategy to enhance the responsiveness of patients treated with immunotherapy [351]. 

## 6. Evolution of the Landscape on Cancer Neoepitopes during Immunotherapy

In cancers, approximately 99% of somatic substitutions are well tolerated and accumulate in malignant cells, often leading to hypermutation [352,353]. Prediction models estimate TNA numbers to be associated with mutational load; but experimental validation reveals that only a small fraction of neoepitopes can bind to MHC, recognized by TCR and be immunogenic [354]. The highly immunogenic TNAs generated by nonsynonymous mutations are selectively depleted by the host immune surveillance thereby shaping tumor evolution [355,356]. A model for evolution of Tumor-Immune associations proposes that tumor intrinsic factors like TNAs elicit immune infiltrates which kill immunogenic clones; driving the growth of immune resistant or immune suppressing subclones [356] (Figure 3). 

Studies show that the TNA landscape evolves heterogeneously through multiple distinct tumor immune microenvironments, such as in metastatic lesions, over the course of tumor progression and treatment status [357,358,359]. Furthermore, in a case of long term cancer survivors, neoantigen quality rather than quantity is identified as a biomarker of immunogenic tumors, that could be used to better direct immunomodulatory treatments [313]. Moreover, the number of TNAs per missense mutation, referred to as neoantigen frequency but not the number of missense mutations or total TNAs, correlates with clinical outcomes and could act as a prognostic factor and potential biomarker for cancer immunotherapy [360]. Tumor heterogeneity seems to favor TNA diversity; in addition to high clonal TNA burden, tumors appear to respond better to immune checkpoint blockers and have improved prognosis compared to low clonal TNA bearing tumors [314,361,362].

Despite the significant contribution of immune checkpoint blockers in cancer immunotherapy, during immune checkpoint blockade, the dynamics of mutational landscapes affect tumor neoantigens through genomic changes to truncal and subclonal mutations that eliminate immunogenic TNAs and develop clones with acquired resistance, further complicating cancer treatment [307,363]. In addition, immune checkpoint blockers are found to exert T cell-dependent immunoselective pressure in tumor progression, effectively potentiating cancer immunoediting [308,364]. 

Tumor and microenvironment changes are observed in response to anti-PD-1 therapy. Responding patients exhibit reduction in mutation and neoantigen burden as well as clonal evolution-directed immunoediting [365]. Furthermore, expansion of the T cell repertoire and production of specific T cell clonotypes target tumor neoantigens during anti-PD-1 treatment, which also appears to upregulate an array of immune checkpoint-related genes [365]. Moreover, immunotherapy with anti-CTLA-4 antibodies seems to enhance T cell priming and induce newly detected T cell responses broadening the TCR repertoire [366,367]. Mobilization and increase of the TCR repertoire is also observed after immunotherapy with anti-CD4 monoclonal antibody or TIL and is associated with increased antitumor immunity and improved treatment response [368,369,370].

Strategies implementing longitudinal and multiregional sampling of tumors throughout cancer progression and treatment of individual patients provide the best information of tumor neoantigen and microenvironment evolution [326]. Interestingly, despite the huge challenges, researchers were able to investigate tumor response to immune checkpoint blockers over time and identified potential mechanisms of therapeutic resistance as well as adaptive immune signatures on early treated biopsies that predict response to immune checkpoint blockers [371,372].

## 7. Conclusions

In summary, the immunotherapeutic developments during the last years have significantly increased our hopes for successfully treating different cancer types. However, the development of new, more effective anticancer immunotherapeutic agents, urges a thorough understanding of the aspects that allow cancer cells to escape elimination by immune cells. Many cancer patients have shown a better clinical response when treated with a single of the combined immunotherapies. Still, a broader group of patients needs to benefit from these immunotherapies. Furthermore, we need to keep in mind that these immunotherapies do not come without adverse effects and complications. Therefore, the focus has already shifted towards the design of novel immunotherapies, which are tailored to the patient’s genetic profile and the appropriate selection of cancer patients who are more likely to present durable responses.

## Figures and Tables

**Table 1:** Recent advances in cancer immunotherapy.

Immune Checkpoint Blockade	Cancer	Organism	References
Ipilimumab (anti–CTLA-4)	Melanoma	Humans	[3]
Tremelimumab (anti–CTLA-4)	Hepatocellular carcinoma (HCC)	Humans	[13]
Atezolizumab (anti-PD-L1)	Bladder, NSCLC	Humans	[14,15]
Avelumab (anti-PD-L1)	Merkel cell carcinoma	Humans	[16]
Pembrolizumab (anti-PD-1)	NSCLC, Melanoma, cHL, RCC, HNSCC, dMMR or MSI+ tumors	Humans	[17]
Nivolumab (anti-PD-1)		Humans	[18]
Durvalumab (anti-PD-L1)	NSCLC, Urothelial carcinoma	Humans	[19,20]
IDO5 (IDO inhibitor)	NSCLC	Humans	[21]
1-L-MT (IDO1 inhibitor)	Mastocytoma, CRC	Mice, cell lines	[22,23]
Indoximod (IDO1 inhibitor)	Melanoma, Prostate, Brain, AML	Humans	[24,25,26,27]
680C91 & LM10 (TDO inhibitors)	Various cancer types	Mice, cell lines	[28]
Navoximod (IDO1 inhibitor)	Advanced solid tumors	Humans	[29,30]
Epacadostat (IDO1 inhibitor)	Multiple advanced solid tumors	Humans	[31]
Samalizumab (anti-CD200)	Bladder carcinoma	Humans	[32]
Varlilumab (anti-CD27)	Advanced refractory solid tumors	Humans	[33,34]
KWAR23 (anti-SIRPa)	Burkitt’s lymphoma, RCC, melanoma	Mice, cell lines	[35,36]
Urelumab (anti-CD137)	CRC, Gastric, Lymphoma	Mice, Humans	[37,38]
Lirilumab (anti-KIR2D mAb)	HNSCC, Lymphoma, myeloid malignancies	Humans, Mice, cell lines	[39,40,41]
**Cytokine Therapy**			
Interleukin-2 (IL-2)	Melanoma, kidney, polycythemia vera	Humans	[42,43,44]
Interferon alpha (IFN-α)	Melanoma	Humans	[45]
**Cellular Therapy**			
Tisagenlecleucel (anti-CD19)	NHL, ALL, DLBCL	Humans	[46,47]
Axicabtagene ciloleucel (anti-CD19)	Large B-Cell Lymphoma, NHL	Humans	[48,49]
anti-MUC1 CAR-T cells	Seminal vesicle carcinoma	Humans	[50]
CD33 knockout hematopoietic stem and progenitor cells (HSPCs)	Acute myeloid leukemia (AML)	Macaques, mice	[51]
Tumor antigen-loaded dendritic cells	Renal cell carcinoma (RCC)	Cell lines	[52]
IL-12p70-producing DCs	Melanoma	Humans	[53]
coTCRcys-transduced T cells	Nasopharyngeal	Cell lines	[54]
**Therapeutic Vaccines**			
Hepatitis B virus (HBV)	HCC	Humans	[55]
Human papilloma virus (HPV)	Cervical, HNSCC, Oropharyngeal	Humans	[56,57,58]
Sipuleucel-T	Prostate	Humans	[59]
anti-gp100	Melanoma	Humans	[60]
STINGVAX & anti–PD-1(G4)	Melanoma, Pancreatic, Colon, Tongue	Mice	[61]
GVAX & CRS-207	Pancreatic	Humans	[62]

**Table 2:** Co-stimulatory and co-inhibitory markers in cancer immunity.

Immune Receptors	Cancer Cell or APC	T, Treg, M, NK Cells	References
**co-stimulatory**	CD80/86 (B7.1/2)	CD28	[87]
	4-1BBL	CD137 (4-1BB)	[88]
	OX-40L	OX40	[89]
	CD70	CD27	[90]
	ICOSL (B7RP1)	ICOS	[91]
	GITRL	GITR	[92]
	B7-H7 (HHLA2)	TMIGD2 (CD28H)	[93]
	LIGHT	HVEM (CD270)	[94]
	CD40	CD40L	[95]
	PVR (CD155)	DNAM-1 (CD226)	[96]
	CD48	2B4 (CD244)	[97]
	CD47	SIRPa	[98]
	MHC-I	CD94/NKG2	[99]
	LFA3 (CD58)	CD2	[100]
	ICAM	LFA1	[101]
	MHC-I or II	TCR/CD3	[102,103]
**co-inhibitory**	CD80/86 (B7.1/2)	CTLA-4 (CD152)	[104]
	PD-L1/2	PD-1	[105]
	PD-L1	B7-1 (CD80)	[106]
	IDO1/2, TDO	Tryptophan	[22,107]
	HVEM (CD270)	BTLA, CD160	[108]
	GAL9, PtdSer, HMGB1, Ceacam-1	TIM3	[109,110]
	PVR (CD155)	CD96, TIGIT, DNAM-1	[111,112]
	PVRL2 (CD112)	CD112R, TIGIT	[113,114]
	Adenosine	A2aR	[115]
	CD200	CD200R	[116]
	B7-H3 (CD276)	?, IL20RA	[117,118]
	B7-H4 (B7S1, VTCN1)	?	[119]
	B7-H5 (VISTA)	?, VSIG-3	[120,121]
	B7-H7 (HHLA2)	?	[122]
	MHC-I	KIR	[123]
	MHC-I or II, FGL1	LAG-3	[124,125]

APC, antigen presenting cell; Treg, T regulatory cell; M, Macrophage; NK cell, natural killer cell.
